# A Critical Perspective on the Supplementation of *Akkermansia muciniphila*: Benefits and Harms

**DOI:** 10.3390/life13061247

**Published:** 2023-05-24

**Authors:** Vito Chiantera, Antonio Simone Laganà, Sabrina Basciani, Maurizio Nordio, Mariano Bizzarri

**Affiliations:** 1 Unit of Gynecologic Oncology, ARNAS “Civico—Di Cristina—Benfratelli”, Department of Health Promotion, Mother and Child Care, Internal Medicine and Medical Specialties (PROMISE), University of Palermo, 90127 Palermo, Italy; 2 Department of Experimental Medicine, Section of Medical Pathophysiology, Food Science and Endocrinology, Sapienza University of Rome, 00161 Rome, Italy; 3 Department of Experimental Medicine, Sapienza University of Rome, 00161 Rome, Italy; 4 System Biology Group Laboratory, Sapienza University, 00161 Rome, Italy

**Keywords:** *Akkermansia muciniphila*, benefits, harms, microbiota, IBD, PCOS, endometriosis

## Abstract

*Akkermansia muciniphila* is a mucin-degrading bacterium of the intestinal niche, exerting beneficial effects on the host metabolic profile. Accumulating evidence indicated *Akkermansia* as a promising therapeutic probiotic against metabolic disorders such as obesity, type 2 diabetes and cardiovascular diseases. However, in specific intestinal microenvironments, its excessive enrichment may be not beneficial. Conditions like inflammatory bowel disease (IBD), *Salmonella typhimurium* infection or post-antibiotic reconstitution may not benefit from *Akkermansia* supplementation. Furthermore, using *Akkermansia* in patients with endocrine and gynecological disorders—such as polycystic ovary syndrome (PCOS) or endometriosis—that have a higher risk of developing IBD, should be critically evaluated. In addition, a cautionary note comes from the neurological field, as the gut microbiota of patients suffering from Parkinson’s disease or multiple sclerosis exhibits a characteristic signature of *Akkermansia municiphila* abundance. Overall, considering these controversial points, the use of *Akkermansia* should be evaluated on an individual basis, avoiding risking unexpected effects.

## 1. Introduction

*Akkermansia muciniphila* is a commensal bacterium of the intestinal niche first isolated in 2004 by Derrien and colleagues [1]. Since then, researchers and physicians have increased their interest on such microbe, especially in recent years, as it has been emerging as “a promising next-generation beneficial microbe” due to its role in maintaining host wellbeing with a particular regard for the management of metabolic diseases [2].

In detail, *Akkermansia* is an oval-shaped, anaerobic Gram-negative bacterium, representing about 3% of the gut microbial community. It is the only representative member of the *Verrucomicrobia* phylum found in mammal gastrointestinal samples, and its abundance gradually increases throughout the life course [3,4]. It starts colonizing the intestinal tract early, and from the first year of life, its levels become equal to those observed in healthy adults, and then decrease in the elderly.

The core of its life cycle and metabolism relies on the process of degrading mucin as a source of carbon, nitrogen and energy, thanks to several mucolytic enzymes encoded by its genome (glycosyl hydrolases, proteases, sulfatases, and sialidases). Mucin is secreted by the glandular epithelium of the gastro-intestinal tract and represents the main glycoprotein of the mucus. It plays a crucial role in physical protection as well as in regulating the passage of water within the gut, along with ions and immune mediators such as antimicrobial peptides and immunoglobulin-A [5,6]. In addition, mucin also acts as the first line of defense against mechanical damage, pathogen invasion and toxins, providing a surface layer to bacteria for their growth, adhesion and protection [7,8]. Therefore, the process of mucin degradation by *Akkermansia* needs to be highly regulated. Indeed, the activity of degrading mucin stimulates mucosa to produce new mucus, thus strengthening the epithelial barrier, but the excessive degradation may induce a severe susceptibility to pathogens, inflammatory intestinal diseases and colorectal cancer. Moreover, at the same time, an excessive presence of mucus may be symptomatic of mucosal inflammatory processes.

The scientific interest on *Akkermansia muciniphila* arises from evidence highlighting that its abundance in the gut correlates with host health, while its alterations with several dysfunctions [9,10,11]. This is the case of metabolic diseases including obesity, type 2 diabetes mellitus (T2DM), cardiovascular diseases (CVD) and non-alcoholic fatty liver disease (NAFLD), in which the levels of *Akkermansia muciniphila* decrease. However, at the same time, an excessive enrichment in *Akkermansia muciniphila*, in specific intestinal microenvironments, may exacerbate local inflammation caused by damages on the epithelial barrier [12,13,14]. For instance, increasing *Akkermansia* in a condition of *Salmonella typhimurium* infection or in a condition of intestinal bowel diseases could have no expected beneficial effects [14,15].

In addition, a cautionary note on its wide use has come from the neurologic field since in some pathological contexts, including Parkinson’s disease (PD) and multiple sclerosis (MS), the intestinal microbiota exhibits a characteristic signature of *Akkermansia municiphila* abundance [16].

Therefore, in light of these controversial points, evaluating the use of *Akkermansia* on an individual basis should be critically considered.

## 2. Regulation of the Host Metabolic Profile: The Benefits

Several pieces of evidence refer to *Akkermansia muciniphila* as a promising therapeutic agent with a probiotic role and several metabolic applications [17]. Indeed, both pre-clinical and clinical studies demonstrated its efficacy in improving the clinical picture of metabolic syndrome and obesity with beneficial effects on insulin sensitivity, lipoprotein metabolism and hepatic metabolic inflammation [18,19,20]. A recent review focused on the correlation between gut microbiota and intestinal homeostasis by exploring the involvement of *Akkermansia* in the development of metabolic disorders and its role in the maintenance of intestinal health and host metabolic modulation [21]. *Akkermansia muciniphila* is highly present in the intestinal microbiota of healthy individuals: its levels correlate with body weight and its supplementation may induce preventive and therapeutic effects against metabolic dysfunctions [2,22,23].

The best-described effects of *Akkermansia* regard its ability to strengthen the integrity of the intestinal barrier, modulate insulin resistance and protect from metabolic inflammation. A study by Reunanen et al. found that *Akkermansia* could adhere to the intestinal epithelium and enhance the enterocyte monolayer integrity in vitro, suggesting its ability to enhance the thickness of the mucus layer and repair the damaged gut barrier [24].

Preclinical studies on murine models of obesity revealed that such beneficial effects rely on its ability to increase (i) the expression of tight junction proteins; (ii) the number of goblet cells, which are specialized epithelial cells secreting mucin and creating a protective mucus layer; (iii) the thickness of the mucus layer.

*Akkermansia* also helps to preserve the epithelial barrier’s integrity by stimulating anti-inflammatory pathways [9,25,26]. In fact, from the fermentation of mucin, *Akkermansia muciniphila* produces short chain fatty acids (SCFAs) such as acetate and propionate [1], thus improving intestinal integrity and reducing endotoxemia [25] arising, for instance, from a condition of obesity [27]. *Akkermansia* also participates in the host immune regulation: a preclinical study on obese mice demonstrated that *Akkermansia* may improve glucose tolerance and attenuate adipose inflammation by inducing Foxp3, which is the lineage specification factor of regulatory T cells [28] that are involved in regulating the immune response to self-antigens, allergens, commensal microbiota as well as infectious agents and tumors [29].

Noteworthy such beneficial effects are not related only to live *Akkermansia muciniphila*, but also to pasteurized *Akkermansia*. The latter may enhance the gut barrier’s function and lead to the attenuation of metabolic endotoxemia. A clinical trial by Depommier and colleagues demonstrated that the oral assumption of pasteurized *Akkermansia muciniphila* in overweight or obese individuals significantly ameliorated insulin sensitivity, decreased insulinemia and plasma total cholesterol, and slightly reduced body weight compared to the placebo group. In addition, pasteurized *Akkermansia* also slightly decreased fat mass and hip circumference compared to the baseline [30].

Considering all the evidence, the European Food Safety Authority (EFSA) recently approved the use of pasteurized *Akkermansia muciniphila* as a safe novel food, opening to the possibility of its commercialization as a food supplement.

Overall, the reported studies suggest that *Akkermansia muciniphila* is a promising probiotic strategy for the treatment of metabolic conditions such as obesity and diabetes. However, extending its wide use in clinical practice needs deeper critical considerations and more clinical trials to test and verify its safety and efficacy.

## 3. Evaluating *Akkermansia muciniphila* Supplementation: The Possible Harms

Despite the beneficial effects on the metabolic profile, it is worth noting that in some cases the abundance of *Akkermansia* may be not effective to induce a clinical metabolic improvement, as recently reported in a study on bariatric patients [31]. In such patients with severe obesity, the increased relative abundance of *Akkermansia* observed after bariatric surgery, failed to correlate with improvements in glucose homeostasis compared to the baseline. The authors explained such discrepancy in respect to previous findings in less obese individuals, linking this result to the severity of both obesity and gut microbiota dysbiosis.

Furthermore, in some other conditions, orally supplementing *Akkermansia* may not have the expected effects on intestinal health and clinical conditions, and therefore, considering the safety and efficacy of its widespread application is quite crucial. Indeed, a study by Dingemanse and colleagues demonstrated that in the case of *Salmonella typhimurium* infection, the procolonization of *Akkermansia* makes *Salmonella* a dominant bacterium of microbiota [13,15]. In addition, in a mouse model of intestinal neoplasia, a gavage with *Akkermansia* may influence the development of colorectal cancer by increasing the number and the size of tumors [32].

Inflammatory bowel disease (IBD) is another condition in which the use of *Akkermansia* should be carefully evaluated. In this condition, the gut barrier function is already compromised, and a mucin-degrader probiotic may not be the appropriate choice. This was evidenced in a preclinical study on a mouse model of IBD (IL-10^−/−^), in which the supplementation of *Akkermansia* may be not indicated due to the development of colitis [14]. A condition of prolonged intestinal inflammation, as occurs in the IBD, may be of risk for colonic tumorigenesis, and it is referred to as colitis-associated colorectal cancer (CAC) [33]. Therefore, the risk of exacerbating pathogenic infections and intestinal inflammation in conditions of compromised gut barrier functionality, is a common problem to consider before using mucin-degrading bacteria such as *Akkermansia* [34].

Noteworthy IBD may also often occur in women suffering from endocrine and gynecological disorders such as polycystic ovary syndrome (PCOS) or endometriosis [35]. Although patients with PCOS may exhibit glucose and lipid metabolic alterations, the use of *Akkermansia* needs proper attention in this context. Indeed, these patients may also suffer from dysbiosis of the gut microbiota with chronic intestinal inflammation, which can expose them to a higher risk of developing IBD [36,37]. A longitudinal study found that women with endometriosis may be 80% more likely to develop IBD compared with women without endometriosis [38]. Therefore, considering that patients suffering from PCOS or endometriosis have a higher risk of developing IBD compared to healthy controls, extending the use of *Akkermansia* in the management of such diseases should be carefully evaluated on an individual basis.

The excessive enrichment of *Akkermansia* may alter the process of mucin degradation, thus impairing the intestinal barrier and inducing the secretion of inflammatory cytokines (IL-1β, IL-6, and TNF-α) [12,14,39]. Of course, the mucosal barrier damage and the pro-inflammatory effects of *Akkermansia* are certainly context-dependent [14,40], and studies on the immune-compromised model cannot be directly translated into a human context.

However, considering that other studies also report controversial results in these contexts [41,42], further studies are needed to determine the exact role of *Akkermansia*, live or pasteurized, or its metabolites, in developing colitis and CAC.

The extensive use of *Akkermansia* in the post-antibiotic reconstitution of the microbial community may further make the intestinal barrier’s functionality even worse. A recent study by Wang and colleagues [43] demonstrated that in the particular context of CAC, post-antibiotic *Akkermansia* replenishment exacerbates the intestinal barrier damage and increases colonic and systemic inflammation, thus interfering with the reconstitution of the intestinal microbiota and its metabolic function [44]. Once antibiotic treatments are stopped, the microbiota undergoes a dynamic rebuilding process, which is often slow and incomplete [45,46,47]. Therefore, stimulating such changing microbiota by using probiotics may be not beneficial in this phase. In this scenario, we take the opportunity to remark that probiotics should be used with caution, particularly after antibiotic treatment.

Along with the attention of using *Akkermansia muciniphila* in compromised intestinal conditions, recent findings from the neurologic field indicated a cautionary use of such probiotic. Different studies revealed an increase in *Akkermansia* abundance in patients suffering from Parkinson’s disease (PD) [16,48,49]. The elevated abundance of *Akkermansia* seems to be one of the features of the intestinal microbiota in such patients. In line with this, in 2017, a study found that individuals with rapid eye movement sleep behavior disorder, which is considered a pre-motor symptom of PD, exhibited elevated intestinal *Akkermansia* levels [50]. In addition, other studies revealed an increased intestinal abundance of *Akkermansia* also in subjects with multiple sclerosis (MS) [51,52,53,54]. Different studies demonstrated that transplanting into a mouse model of MS fecal microbiota samples from MS-affected mice, exposed to a worse disease progression compared to transplanting fecal microbiota samples from healthy controls. Although speculative, some authors hypothesized that the activation of the Toll-like Receptor 2 (TLR2) or the modulation of glucose and cholesterol homeostasis [9,25], induced by *Akkermansia*, may determine unexpected deleterious consequences for neurological health in certain individuals. Even though PD and MS all involve the immune system as well as metabolic alterations, to date, no mechanistic studies have deeply explored this association, and evidence underpinning these observations is still lacking.

Another crucial aspect to bear in mind, especially when evaluating the safety of *Akkermansia*, is its evolutionary potential to acquire antimicrobial resistance genes (ARGs) under antibiotic selective pressure [55,56,57]. *Akkermansia* is gaining significant attention for its potential application in food supplements and pharmaceutical formulations as well as other anaerobic gut commensals associated with human health (*Bacteroides* spp., *Clostridium butyricum*, *Faecalibacterium prausnitzii*), however, all these genera and microbial species do not have a history of safe use yet [58,59]. With the introduction of *Akkermansia muciniphila* in the food chain, the evaluation of the antimicrobial susceptibility of this bacterium becomes fundamental to meet the safety recommendations of EFSA. Phenotypic tests have confirmed the antibiotic resistance profile of some strains of *Akkermansia*; however, further studies involving a larger number of *Akkermansia* strains are necessary to demonstrate the safety of this microbial species, considering that the coexistence of several microbial populations in the gut provides ideal conditions for gene exchange [56].

## 4. Conclusions

Considering all the benefits and harms, the supplementation of *Akkermansia muciniphila* should be critically evaluated. Although it has extensive and well-proven positive effects on metabolic profiles, some different behaviors have questioned its beneficial clinical effects, as in the case of bariatric patients. In addition, some critical questions arise from (i) the use in the context of chronic intestinal inflammation, (ii) the neurologic field and (iii) the evaluation of its safety regarding the potential of carrying antimicrobial resistance. Further studies are necessary to clearly elucidate the fields of application and to delineate the safety of *Akkermansia*, avoiding its use when it is unhelpful or not strictly recommended, as in the case of IBD and related endocrine and gynecological disorders such as PCOS or endometriosis, and in the case of PD and MS. It is worth bearing in mind that the administration of *Akkermansia* should be carefully evaluated on an individual basis, thus tailoring therapies on patients’ clinical conditions. Maintaining the homeostasis of gut microbiota by just providing substrates useful for bacterial proliferation, could be a safe approach without the unexpected effects.

## Data Availability

Not applicable.
